# Supplementary material for: BAGEL: Protein engineering via exploration of an energy landscape
Source: PLoS Comput Biol. 2025 Dec 3;21(12):e1013774. doi: 10.1371/journal.pcbi.1013774 (PMC12688157; doi:10.1371/journal.pcbi.1013774)
Supplement: S1 Text — This file contains information on all the energy terms, weights, and optimization parameters used for the simple peptide binder designs (CA4, EGFR, DERF7), targeting intrinsically disordered epitopes (ASYN, CD28, P53, SUMO1), multi-State selective binder design, and enzyme variant generation. (PDF) [file pcbi.1013774.s001.pdf]

Experimental Details

Here, we detail the exact energy terms and their parameters used for all designs presented in the main text. While the Monte Carlo sampling procedure is inherently stochastic, using these settings with `BAGEL` v0.1 will yield qualitatively similar results across repeated runs. We do not specify the number of tempering cycles (n_{cycles}) used in `SimulatedTempering`, as the optimal number of cycles depends strongly on the initial random candidate sequence and the specifics of the design task. In practice, n_{cycles} should be tuned to ensure sufficient exploration. An example input file for the simple peptide binder case is also provided.

Parameters - Simple Peptide Binder

Table 1: Design parameters for the CA4 peptide binder. Groups are G_{target} (therapeutic target), G_{hotspot} (binding interface), and G_{binder} (designable peptide). All `EnergyTerms` use `ESMFold` as the `Oracle`.

**Protein**	**Description**	**Residue Group**	**Value / Definition**
Target	UniProt ID	G_{target}	P22748 (CAH4_HUMAN), all residues
	Hot-spot residues	G_{hotspot}	Glu2–Val9; Val65–Asn78; Ala93–His99; Glu120–His125; Ile145–Val147; Leu206–Val216
Binder	Length	G_{binder}	30 aa (all mutable)
**`State`**	**`EnergyTerm`**	**Affected Group**	**Details**
bound	`PTMEnergy`	$G_{\text{target}} \cup G_{\text{binder}}$	$w = 1.0$
	`OverallPLDDTEnergy`	$G_{\text{target}} \cup G_{\text{binder}}$	$w = 1.0$
	`PLDDTEnergy`	G_{binder}	$w = 2.0$
	`HydrophobicEnergy`	$G_{\text{target}} \cup G_{\text{binder}}$	$w = 1.0$, all atoms considered (`surface_only=False`)
	`PAEEnergy`	$G_{\text{hotspot}} \leftrightarrow G_{\text{binder}}$	$w = 6.0$, interface PAE
	`SeparationEnergy`	$G_{\text{hotspot}} \leftrightarrow G_{\text{binder}}$	$w = 1.0$
**`Minimizer`**	**Parameter**	**Value**	
`SimulatedTempering`	`MutationProtocol`	`Canonical`	
	n_{mut}	1	
	T_{low}	0.1	
	T_{high}	1.0	
	$n_{\text{low, steps}}$	400	
	$n_{\text{high, steps}}$	100	
	$n_{\text{best system}}$	500	

Table 2: Design parameters for the EGFR peptide binder. Groups are G_{target} (therapeutic target), G_{hotspot} (binding interface), and G_{binder} (designable peptide). All `EnergyTerms` use `ESMFold` as the `Oracle`.

**Protein**	**Description**	**Residue Group**	**Value / Definition**
Target	UniProt ID	G_{target}	P00533 (EGFR), only residues Cys305 to Val482
	Hot-spot residues	G_{hotspot}	Gly354–Thr358; Gly435–Val437; Ile439–Gly441
Binder	Length	G_{binder}	30 aa (all mutable)
**`State`**	**`EnergyTerm`**	**Affected Group**	**Details**
bound	`PTMEnergy`	$G_{\mathrm{target}} \cup G_{\mathrm{binder}}$	$w = 1.0$
	`PLDDTEnergy`	G_{binder}	$w = 2.0$
	`HydrophobicEnergy`	$G_{\mathrm{target}} \cup G_{\mathrm{binder}}$	$w = 1.0$, all atoms considered (`surface_only=False`)
	`PAEEnergy`	$G_{\mathrm{hotspot}} \leftrightarrow G_{\mathrm{binder}}$	$w = 4.0$, interface PAE
	`SeparationEnergy`	$G_{\mathrm{hotspot}} \leftrightarrow G_{\mathrm{binder}}$	$w = 0.2$
**`Minimizer`**	**Parameter**	**Value**	
`SimulatedTempering`	`MutationProtocol`	`Canonical`	
	n_{mut}	1	
	T_{low}	0.001	
	T_{high}	1.0	
	$n_{\text{low, steps}}$	400	
	$n_{\text{high, steps}}$	100	
	$n_{\text{best system}}$	500	

Table 3: Design parameters for the DERF7 peptide binder. Groups are G_{target} (therapeutic target), G_{hotspot} (binding interface), and G_{binder} (designable peptide). All `EnergyTerms` use `ESMFold` as the `Oracle`.

**Protein**	**Description**	**Residue Group**	**Value / Definition**
Target	UniProt ID	G_{target}	Q26456 (ALL7_DERFA)
Binder	Length	G_{binder}	30 aa (all mutable)
**`State`**	**`EnergyTerm`**	**Affected Group**	**Details**
bound	`PTMEnergy`	$G_{\mathrm{target}} \cup G_{\mathrm{binder}}$	$w = 1.0$
	`OverallPLDDTEnergy`	$G_{\mathrm{target}} \cup G_{\mathrm{binder}}$	$w = 1.0$
	`PLDDTEnergy`	G_{binder}	$w = 2.0$, confident binder
	`HydrophobicEnergy`	$G_{\mathrm{target}} \cup G_{\mathrm{binder}}$	$w = 1.0$, all atoms considered (`surface_only=False`)
	`PAEEnergy`	$G_{\mathrm{target}} \leftrightarrow G_{\mathrm{binder}}$	$w = 6.0$, interface PAE
	`SeparationEnergy`	$G_{\mathrm{target}} \leftrightarrow G_{\mathrm{binder}}$	$w = 1.0$
**`Minimizer`**	**Parameter**	**Value**	
`SimulatedTempering`	`Mutator`	`Canonical`	
	n_{mut}	1	
	T_{low}	0.1	
	T_{high}	1.0	
	$n_{\text{low, steps}}$	400	
	$n_{\text{high, steps}}$	100	
	$n_{\text{best system}}$	500	

Parameters - Target Intrinsically Disordered Epitopes

Table 4: Design parameters for the ASYN peptide binder. Groups are G_{target} (therapeutic target), G_{hotspot} (targeted disordered epitope), and G_{binder} (designable peptide). All `EnergyTerms` use `ESMFold` as the `Oracle`.

**Protein**	**Description**	**Residue Group**	**Value / Definition**
Target	UniProt ID	G_{target}	P37840 (SYUA_HUMAN)
	Hot-spot residues	G_{hotspot}	Leu100–Ala140
Binder	Length	G_{binder}	50 aa (all mutable)
**`State`**	**`EnergyTerm`**	**Affected Group**	**Details**
bound	PTMEnergy	$G_{\mathrm{target}} \cup G_{\mathrm{binder}}$	$w = 1.0$
	OverallPLDDTEnergy	$G_{\mathrm{target}} \cup G_{\mathrm{binder}}$	$w = 1.0$
	PLDDTEnergy	G_{binder}	$w = 4.0$, confident binder
	PLDDTEnergy	G_{hotspot}	$w = 4.0$, induce order on epitope
	HydrophobicEnergy	$G_{\mathrm{target}} \cup G_{\mathrm{binder}}$	$w = 2.0$, all atoms considered (`surface_only=False`)
	PAEEnergy	$G_{\mathrm{hotspot}} \leftrightarrow G_{\mathrm{binder}}$	$w = 4.0$, interface PAE
	SeparationEnergy	$G_{\mathrm{hotspot}} \leftrightarrow G_{\mathrm{binder}}$	$w = 0.2$
**`Minimizer`**	**Parameter**	**Value**	
`SimulatedTempering`	`Mutator`	`Canonical`	
	n_{mut}	1	
	T_{low}	0.2	
	T_{high}	2.0	
	$n_{\text{low, steps}}$	50	
	$n_{\text{high, steps}}$	50	
	$n_{\text{best system}}$	100	

Table 5: Design parameters for the CD28 peptide binder. Groups are G_{target} (therapeutic target), G_{hotspot} (targeted disordered epitope), and G_{binder} (designable peptide). All `EnergyTerms` use `ESMFold` as the `Oracle`.

**Protein**	**Description**	**Residue Group**	**Value / Definition**
Target	UniProt ID	G_{target}	P10747 (CD28_HUMAN)
	Hot-spot residues	G_{hotspot}	Gly121–Arg140
Binder	Length	G_{binder}	30 aa (all mutable)
**`State`**	**`EnergyTerm`**	**Affected Group**	**Details**
bound	PTMEnergy	$G_{\mathrm{target}} \cup G_{\mathrm{binder}}$	$w = 1.0$
	OverallPLDDTEnergy	$G_{\mathrm{target}} \cup G_{\mathrm{binder}}$	$w = 1.0$
	PLDDTEnergy	G_{binder}	$w = 4.0$, confident binder
	PLDDTEnergy	G_{hotspot}	$w = 4.0$, induce order on epitope
	HydrophobicEnergy	$G_{\mathrm{target}} \cup G_{\mathrm{binder}}$	$w = 2.0$, all atoms considered (`surface_only=False`)
	PAEEnergy	$G_{\mathrm{hotspot}} \leftrightarrow G_{\mathrm{binder}}$	$w = 4.0$
	SeparationEnergy	$G_{\mathrm{hotspot}} \leftrightarrow G_{\mathrm{binder}}$	$w = 0.2$
	SecondaryStructureEnergy	G_{hotspot}	$w = 8.0$, alpha-helix
**`Minimizer`**	**Parameter**	**Value**	
`SimulatedTempering`	`Mutator`	`Canonical`	
	n_{mut}	1	
	T_{low}	0.3	
	T_{high}	2.0	
	$n_{\text{low, steps}}$	100	
	$n_{\text{high, steps}}$	100	
	$n_{\text{best system}}$	200	

Table 6: Design parameters for the P53 peptide binder. Groups are G_{target} (therapeutic target), G_{hotspot} (targeted disordered epitope), and G_{binder} (designable peptide). All `EnergyTerms` use `ESMFold` as the `Oracle`.

**Protein**	**Description**	**Residue Group**	**Value / Definition**
Target	UniProt ID	G_{target}	P04637 (P53_HUMAN)
	Hot-spot residues	G_{hotspot}	Pro1–Phe18
Binder	Length	G_{binder}	30 aa (all mutable)
**`State`**	**`EnergyTerm`**	**Affected Group**	**Details**
bound	PTMEnergy	$G_{\mathrm{target}} \cup G_{\mathrm{binder}}$	$w = 1.0$
	OverallPLDDTEnergy	$G_{\mathrm{target}} \cup G_{\mathrm{binder}}$	$w = 1.0$
	PLDDTEnergy	G_{binder}	$w = 4.0$, confident binder
	PLDDTEnergy	G_{hotspot}	$w = 4.0$, induce order on epitope
	HydrophobicEnergy	$G_{\mathrm{target}} \cup G_{\mathrm{binder}}$	$w = 2.0$, all atoms considered (`surface_only=False`)
	PAEEnergy	$G_{\mathrm{hotspot}} \leftrightarrow G_{\mathrm{binder}}$	$w = 5.0$, interface PAE
	SeparationEnergy	$G_{\mathrm{hotspot}} \leftrightarrow G_{\mathrm{binder}}$	$w = 0.2$
**`Minimizer`**	**Parameter**	**Value**	
`SimulatedTempering`	`Mutator`	`Canonical`	
	n_{mut}	1	
	T_{low}	0.1	
	T_{high}	1.0	
	$n_{\text{low, steps}}$	200	
	$n_{\text{high, steps}}$	200	
	$n_{\text{best system}}$	400	

Table 7: Design parameters for the SUMO1 peptide binder. Groups are G_{target} (therapeutic target), G_{hotspot} (targeted disordered epitope), and G_{binder} (designable peptide). All `EnergyTerms` use `ESMFold` as the `Oracle`.

**Protein**	**Description**	**Residue Group**	**Value / Definition**
Target	UniProt ID	G_{target}	P63165 (SUMO1_HUMAN)
	Hot-spot residues	G_{hotspot}	Gly1–Gly21
Binder	Length	G_{binder}	30 aa (all mutable)
**`State`**	**`EnergyTerm`**	**Affected Group**	**Details**
bound	PTMEnergy	$G_{\mathrm{target}} \cup G_{\mathrm{binder}}$	$w = 1.0$
	OverallPLDDTEnergy	$G_{\mathrm{target}} \cup G_{\mathrm{binder}}$	$w = 1.0$
	PLDDTEnergy	G_{binder}	$w = 4.0$, confident binder
	PLDDTEnergy	G_{hotspot}	$w = 4.0$, induce order on epitope
	HydrophobicEnergy	$G_{\mathrm{target}} \cup G_{\mathrm{binder}}$	$w = 2.0$, all atoms considered (`surface_only=False`)
	PAEEnergy	$G_{\mathrm{hotspot}} \leftrightarrow G_{\mathrm{binder}}$	$w = 5.0$, interface PAE
	SeparationEnergy	$G_{\mathrm{hotspot}} \leftrightarrow G_{\mathrm{binder}}$	$w = 0.2$
**`Minimizer`**	**Parameter**	**Value**	
`SimulatedTempering`	`Mutator`	`Canonical`	
	n_{mut}	1	
	T_{low}	0.1	
	T_{high}	1.0	
	$n_{\text{low, steps}}$	200	
	$n_{\text{high, steps}}$	200	
	$n_{\text{best system}}$	400	

Parameters - Multi-State Selective Peptide Binder

Table 8: Design parameters for the selective zinc finger binder. Groups are G_{target} (target protein), $G_{\mathrm{non-target}}$ (off-target), G_{hotspot} (target binding interface on target protein), and G_{binder} (designable peptide). All `EnergyTerms` use `ESMFold` as the `Oracle`.

**Protein**	**Description**	**Residue Group**	**Value / Definition**
Target	UniProt ID	G_{target}	P08046 (*Mus musculus*, i.e., mouse variant)
	Hot-spot residues	G_{hotspot}	His81–Asp90, i.e., last 10 residues
Non-target	UniProt ID	$G_{\mathrm{non-target}}$	O00488 (*Homo sapiens*, i.e., human variant)
Binder	Length	G_{binder}	15 aa (all mutable)
**`State`**	**`EnergyTerm`**	**Affected Group**	**Details**
target (binding)	`PTMEnergy`	$G_{\mathrm{target}} \cup G_{\mathrm{binder}}$	$w = 1.0$
	`PLDDTEnergy`	$G_{\mathrm{target}} \cup G_{\mathrm{binder}}$	$w = 1.0$
	`PAEEnergy`	$G_{\mathrm{hotspot}} \leftrightarrow G_{\mathrm{binder}}$	$w = 5.0$
	`SeparationEnergy`	$G_{\mathrm{hotspot}} \leftrightarrow G_{\mathrm{binder}}$	$w = 1.0$
non-target (avoiding)	`PTMEnergy`	$G_{\mathrm{non-target}} \cup G_{\mathrm{binder}}$	$w = 1.0$, remain confident in prediction
	`PLDDTEnergy`	$G_{\mathrm{non-target}} \cup G_{\mathrm{binder}}$	$w = 1.0$, remain confident in prediction
	`PAEEnergy`	$G_{\mathrm{non-target}} \leftrightarrow G_{\mathrm{binder}}$	$w = -5.0$, discourage binding
	`SeparationEnergy`	$G_{\mathrm{non-target}} \leftrightarrow G_{\mathrm{binder}}$	$w = -1.0$, discourage binding
**`Minimizer`**	**Parameter**	**Value**	
`SimulatedTempering`	`Mutator`	`Canonical`	
	n_{mut}	1	
	T_{low}	0.1	
	T_{high}	1.0	
	$n_{\text{low, steps}}$	200	
	$n_{\text{high, steps}}$	50	
	$n_{\text{best system}}$	250	

Parameters - Enzyme Variants with Conserved Active Site

Table 9: Design parameters for the oxidoreductase variant generation. Groups are G_{all} (full enzyme), $G_{\mathrm{conserved}}$ (immutable/conserved residues). The `EmbeddingsSimilarityEnergy` uses the 650M parameter version of `ESM2` as the `Oracle`.

**Protein**	**Description**	**Residue Group**	**Value / Definition**
Target	UniProt ID	G_{all}	P0AEG4 (wild-type oxidoreductase)
	Active Site	$G_{\mathrm{conserved}}$	Cys30, Cys33 (immutable)
**`State`**	**`EnergyTerm`**	**Affected Group**	**Details**
variant	`EmbeddingsSimilarityEnergy`	$G_{\mathrm{conserved}}$	$w = 1.0$, reference: ESM2 embeddings of Cys30 and Cys33 using the original, wild-type sequence
**`Minimizer`**	**Parameter**	**Value**	
`MonteCarloMinimizer`	`Mutator`	`Canonical`	
	n_{mut}	1	
	Temperature	10^{-4}	
	n_{steps}	10,000	
