# Supplementary material for: BAGEL: Protein engineering via exploration of an energy landscape
Source: PLoS Comput Biol. 2025 Dec 3;21(12):e1013774. doi: 10.1371/journal.pcbi.1013774 (PMC12688157; doi:10.1371/journal.pcbi.1013774)
Supplement: S3 Text — (PDF) [file pcbi.1013774.s003.pdf]

Molecular Dynamics Simulations

Below, we present additional *in silico* supporting evidence of the designed sequences presented in the main text. We do not show the supporting data for targeting of the intrinsically disordered epitopes, as that has been already produced in a different manuscript [1].

All simulations were carried out with OpenMM [2], using the Amber ff14SB force field [3] and either explicit [4] or implicit [5] water models. Structures were fixed with `pdbfixer` [2] and minimized with L-BFGS in two phases: first, we restrained the non-hydrogen atoms and minimized the rest of the complex; second, we minimized all atoms together. Equilibration was performed for 10 ns. Both equlibration and production runs were performed in the NVT ensemble. We used cutoffs for non-bonded interactions at 2 nm, solute dielectric of 1.0, solvent dielectric of 78.5, and a timestep of 2 fs. In the explicit solvent simulation, the box is solvated such that there is a padding of 2 nm.

Simple Peptide Binder

The evolution of the number of contacts for the three respective peptide-target complexes is shown in S1 Fig. For both implict and explicit solvent simulations, the binder forms contacts with the target throughout the simulation.

Multi-State Selective Peptide Binder

The evolution of the number of contacts between the binder and the target/off-target is shown in S3 Fig. In the implicit solvent simulations, both form many contacts throughout the 1 µs simulation. Nevertheless, in the explicit solvent simulation, and likely the more accurate system representation, the binder forms contacts with the target (EGR1) throughout the entire 200 ns simulated, while losing contact with the off-target (ZNF) in the first 20 ns. Although it then forms more absolute number of contacts with the off-target than the on-target, the residence time is still much shorter than the on-target (top and middle panels). Moreover, in the hotspot, i.e. the region of the interface between the binder and the target/off-target (defined as the last 10 residues of the sequence), the binder retains contacts, while losing contact with the off-target (bottom panel). This supports the notion of species-selective binding.

Enzyme Variant Generation with Conserved Active Site

We performed molecular dynamics simulations on four diverse DSBA-X variants to assess their structural stability. These variants were selected such that their sequence identity to the wild type (DSBA) is below 40%, and pairwise sequence identity among the variants themselves is also below 40%. S4 Fig shows the Root Mean Square Fluctuations (RMSF) of the four key residues in the active site (Cys30–Cys33). In implicit solvent, the fluctuations of the designed variants are comparable to the wild type. However, the explicit solvent simulations reveal the variants higher RMSF values (around 1.0-1.4 Å) compared to the wild type (around 0.7-0.8 Å). Moreover, S5 Fig shows the distributions of the key residues' Root Mean Square Deviation (RMSD) relative to the wild type ESMFold structure. Similar to S4 Fig, the implicit solvent shows a more conserved dynamic behaviour between the wild type and the variants compared to the explicit-solvent simulations. DSBA-1 shows the closest distribution to DSBA, peaking around 0.8 Å. In contrast, DSBA-3 appears more rigid, with its main peak at a lower RMSD of 0.6 Å. DSBA-2 is markedly different, showing a bimodal distribution with a significant population at a higher RMSD (1.4 Å), indicating it samples conformations further from the reference structure, despite it having a lower RMSF values in the explicit solvent in S4 Fig. This suggests that while some variants maintain the active site structure, others like DSBA-2 may be less structurally conserved. These and other metrics could be used as additional filtering metrics for the designed variants. We do not investigate this further in this manuscript, but note this motivates further development of implementing these metrics into `BAGEL` as energy terms as a function of a molecular dynamics `Oracle`.

References

[1] Jakub Lála, Daniele Visco, and Stefano Angioletti-Uberti. Programming co-folding to design binders for intrinsically disordered epitopes. In *ICLR 2025 Workshop on Generative and Experimental Perspectives for Biomolecular Design*, 2025.

[2] Peter Eastman, Jason Swails, John D. Chodera, Robert T. McGibbon, Yutong Zhao, Kyle A. Beauchamp, Lee-Ping Wang, Andrew C. Simmonett, Matthew P. Harrigan, Chaya D. Stern, Rafal P. Wiewiora, Bernard R. Brooks, and Vijay S. Pande. Openmm 7: Rapid development of high performance algorithms for molecular dynamics. *PLOS Computational Biology*, 13(7):e1005659, July 2017. ISSN 1553-7358. doi:10.1371/journal.pcbi.1005659. URL `http://dx.doi.org/10.1371/journal.pcbi.1005659`.

[3] James A. Maier, Carmenza Martinez, Koushik Kasavajhala, Lauren Wickstrom, Kevin E. Hauser, and Carlos Simmerling. ff14sb: Improving the accuracy of protein side chain and backbone parameters from ff99sb. *Journal of Chemical Theory and Computation*, 11(8):3696–3713, July 2015. ISSN 1549-9626. doi:10.1021/acs.jctc.5b00255. URL `http://dx.doi.org/10.1021/acs.jctc.5b00255`.

[4] Lee-Ping Wang, Todd J. Martinez, and Vijay S. Pande. Building force fields: An automatic, systematic, and reproducible approach. *The Journal of Physical Chemistry Letters*, 5(11):1885–1891, May 2014. ISSN 1948-7185. doi:10.1021/jz500737m. URL `http://dx.doi.org/10.1021/jz500737m`.

[5] Hai Nguyen, Daniel R. Roe, and Carlos Simmerling. Improved generalized born solvent model parameters for protein simulations. *Journal of Chemical Theory and Computation*, 9(4):2020–2034, March 2013. ISSN 1549-9626. doi:10.1021/ct3010485. URL `http://dx.doi.org/10.1021/ct3010485`.
