# Supplementary figures and images for: BAGEL: Protein engineering via exploration of an energy landscape
Source: PLoS Comput Biol. 2025 Dec 3;21(12):e1013774. doi: 10.1371/journal.pcbi.1013774 (PMC12688157; doi:10.1371/journal.pcbi.1013774)

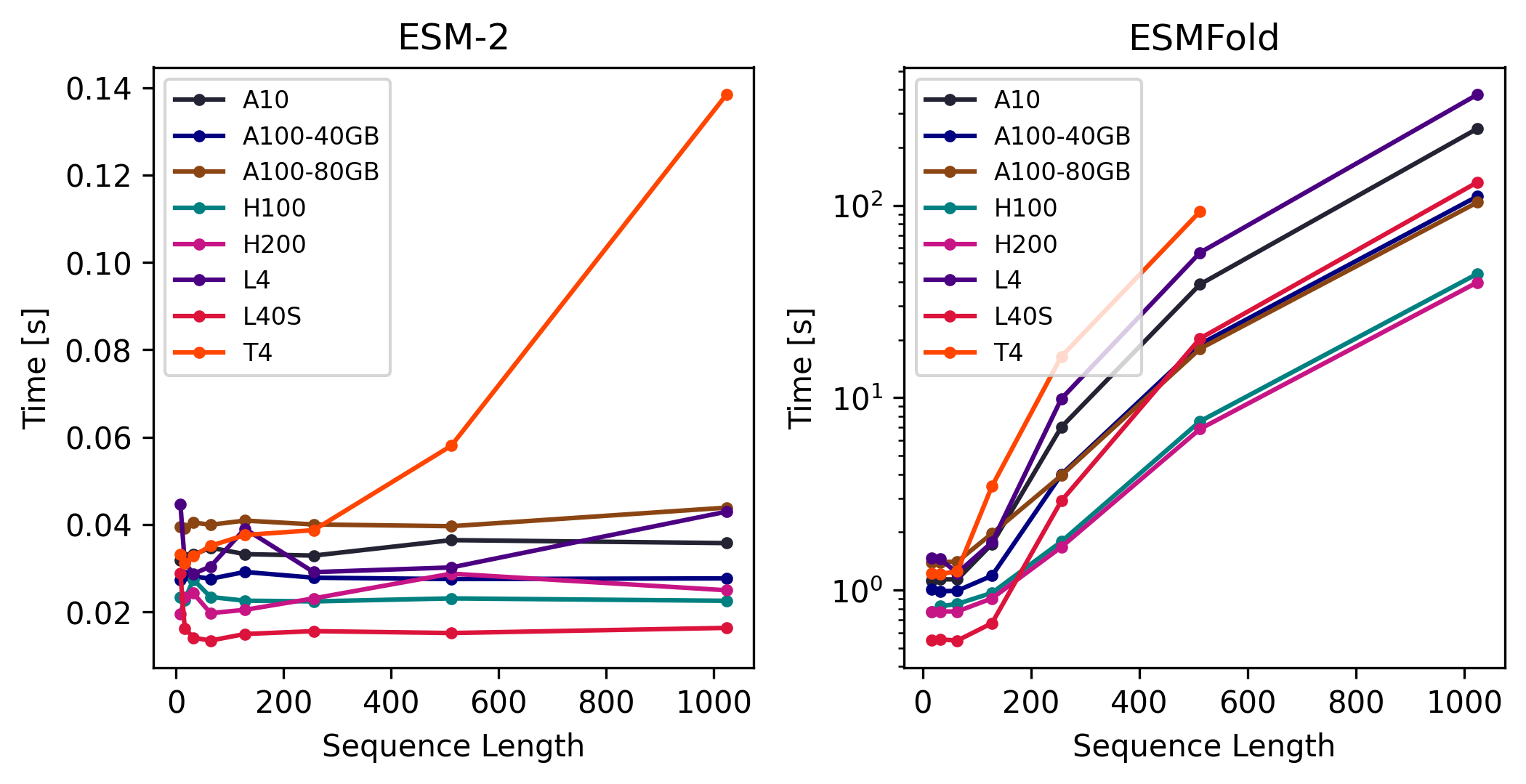

Supplement: S1 Fig — Time taken to infer the embeddings or structure for ESM-2 and ESMFold respectively using various GPUs. The sequence lengths used were [16, 32, 64, 128, 256, 512, 1024] amino acids. (TIF) [file pcbi.1013774.s004.tif]

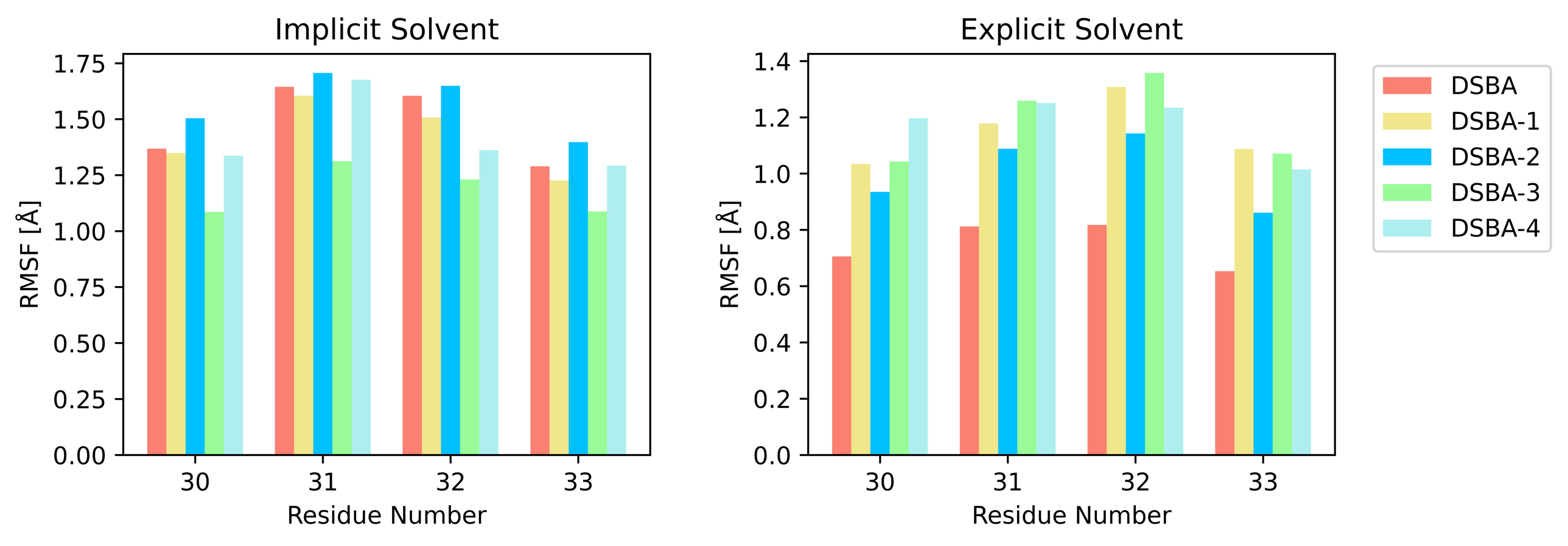

Supplement: S4 Fig — Thermal fluctuations across the four key residues (Cys30–Cys33). In implicit solvent, the designed variants (DSBA-X) show fluctuations comparable to the wild type (DSBA). However, in explicit solvent, all variants exhibit higher RMSF values than the wild type, indicating increased flexibility of the active site relative to the enzyme frame of reference. The Root Mean Square Fluctuations (RMSF) are computed using all the heavy atoms of the four key residues (Cys30–Cys33). The frames of the trajectory were aligned on the Cα atoms of the entire enzyme. All systems were simulated for 650 ns. (TIF) [file pcbi.1013774.s007.tif]

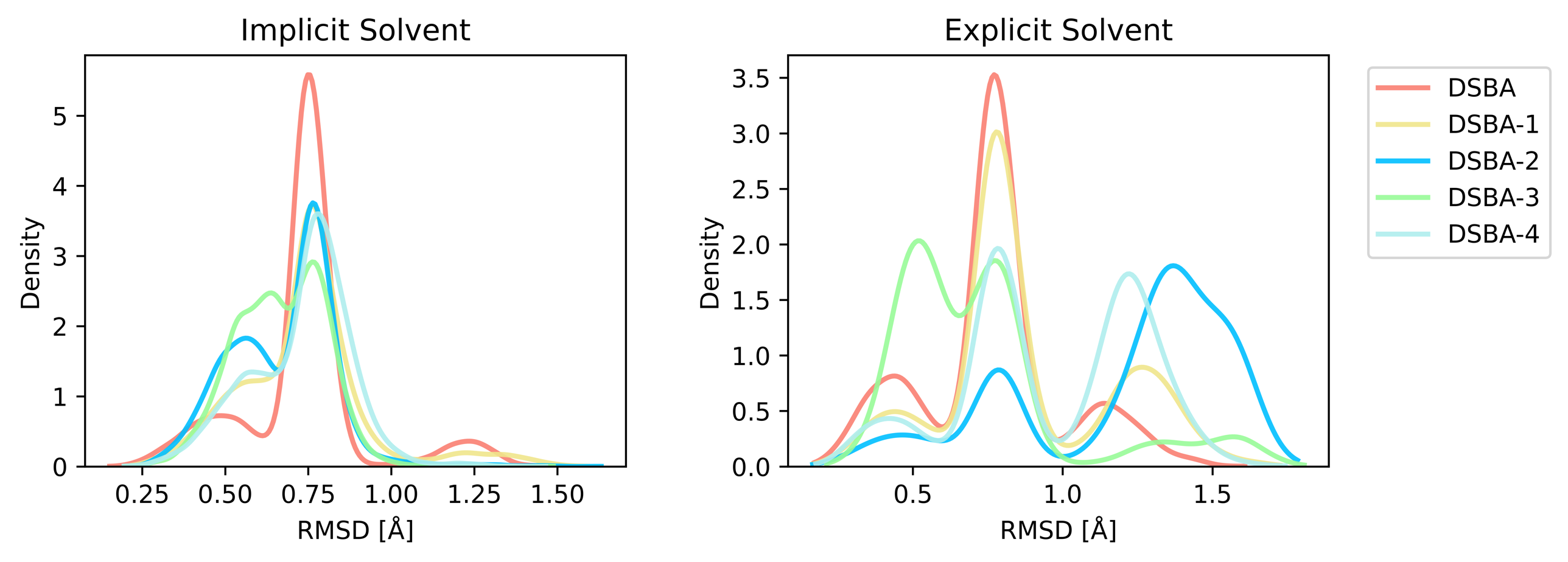

Supplement: S5 Fig — Strucutral deviation of the designed variants and wild type to the wild type ESMFold-derived structure. The implicit solvent show strong agreement, while the explicit solvent shows notable differences. The explicit solvent distributions could be used as a filtering metric for the designed variants. The Root Mean Square Deviation (RMSD)s are computed using all the heavy atoms of the four key residues (Cys30–Cys33). The frames of the trajectory were superimposed to the wild type structure using heavy atoms of the key residues. The distributions are shown as kernel density estimations. All systems were simulated for 650 ns. (TIF) [file pcbi.1013774.s008.tif]
