# Supplementary material for: BAGEL: Protein engineering via exploration of an energy landscape
Source: PLoS Comput Biol. 2025 Dec 3;21(12):e1013774. doi: 10.1371/journal.pcbi.1013774 (PMC12688157; doi:10.1371/journal.pcbi.1013774)
Supplement: Attachment — Submitted filename: Review-SA-BAGEL.pdf [file pcbi.1013774.s009.pdf]

Review

BAGEL: Protein Engineering via Exploration of an Energy Landscape

This manuscript presents BAGEL, a thoughtfully designed, modular framework that redefines computational protein design as a problem of energy landscape exploration rather than purely gradient-based optimization. The work is timely and relevant, addressing long-standing limitations in differentiable, model-specific design workflows. By integrating multi-state optimization, non-differentiable objective handling, and flexible oracle integration, BAGEL represents a substantial conceptual and practical advancement toward a more general, programmable paradigm for protein engineering.

The manuscript succeeds in clearly articulating the motivation and theoretical foundations of BAGEL, as well as demonstrating its versatility across diverse applications from peptide binder design to enzyme variant generation. The model-agnostic philosophy and open-source accessibility are notable strengths, positioning BAGEL as a forward-compatible platform that can evolve alongside emerging deep learning models and molecular predictors.

To further strengthen the work, the following improvements are suggested:

1. The authors must include brief benchmarking results or discussion on runtime scaling, particularly when using large oracles such as AlphaFold or protein language models.
2. The authors should present or reference evaluation metrics (e.g., stability scores, binding energy estimates, or structural RMSDs) in order to better contextualize BAGEL's performance relative to established baselines.

3. Discuss integration and interoperability. basically, explain how BAGEL can complement or interface with other design ecosystems (e.g., Rosetta, PyRosetta, or AlphaFold-Multimer) for broader adoption.
4. Expand briefly on planned or ongoing experimental validations to strengthen confidence in the practical impact of BAGEL-generated designs.
5. Condense some descriptive passages and emphasize unique algorithmic contributions, ensuring that conceptual novelty remains at the forefront.
6. Given that AlphaFold2/3 produce high-accuracy *static* structures, how does the lack of conformational ensemble modeling affect the reliability of designs that depend on flexibility or induced fit?
7. Could integrating molecular dynamics or normal mode analyses into the energy evaluation improve BAGEL's ability to capture dynamic effects?
8. How sensitive is BAGEL's optimization to cases where small conformational shifts dramatically alter binding energetics?
9. To what extent do pLDDT, pTM, or iPAE-based loss terms bias optimization toward regions of the predictor's uncertainty rather than true physical minima
10. Are there strategies in BAGEL to decouple model confidence from energy evaluation for e.g., through ensemble averaging, reweighting, or uncertainty calibration?
11. How might BAGEL handle inconsistent confidence across multiple states or chain interfaces?
12. How does BAGEL handle regions where the oracle's confidence is low? Are there fallback strategies or uncertainty-aware loss terms?
13. Can the framework incorporate experimentally measured kinetic or thermodynamic data to guide multi-state optimization?
14. What is the computational cost of large-scale multi-state runs compared with single-state Monte-Carlo pipelines?

15. How will future diffusion-based or language-model predictors (e.g., AlphaFold-3) be integrated without redesigning the oracle interface?
16. How does BAGEL's multi-state formalism handle coupling between shared chains across different states without double-counting energies?
17. The additive N-body energy formulation mirrors molecular mechanics approaches. How are weights w_{js}calibrated to avoid dominance by any single energy term?
18. Can BAGEL serve as a benchmarking framework to evaluate the robustness of new oracles or differentiable predictors?
19. The notation is occasionally overloaded or ambiguous. For instance, the use of {C}S and {C} could be more consistently distinguished ; sometimes {C}S refers to chains in a particular state, and sometimes to their sequences.

    a. Equation (1) defines Ω = ({C}, {S}), which conceptually conflates the system and its states. A more precise statement might specify Ω as a set of states, each defined by its constituent chains and energy functions:

    $$\Omega = \{S_i = (\{C\}_{S_i}, E_{S_i})\}$$

    b. Summation indices (e.g., over S, j, or s) should be clearly delimited; currently, they shift between symbolic and index notation.

20. While defining three types of oracles (folding, embedding, and property) is comprehensive, the notation for outputs (e.g., y_s, $\vec{z}$, r) could be standardized. It would help to specify how these oracle outputs are integrated into ε; are ε functions differentiable ? Or do they operate as heuristic scoring functions? This distinction affects both theoretical guarantees and computational tractability.

.

21.The MCMC section could more explicitly describe the mutation operator M**,** e.g., whether it performs single-residue substitutions, insertions/deletions, or compositional sampling across multiple chains.

c. The temperature schedule T_n is mentioned but not defined; providing a concrete annealing scheme (e.g., exponential or logarithmic) would make the procedure more actionable.

d. The convergence or stopping criteria ("system's energy reaches a plateau") could be better formalized, perhaps via a threshold on ΔE variance or effective sample size.

22. Some equations (especially Eq. 2–4) could be formatted more clearly with alignment and consistent subscripts.

23. What mechanisms are in place (documentation, API standardization, model registry) to facilitate external contributions and ensure reproducibility?
