# Supplementary material for: BAGEL: Protein engineering via exploration of an energy landscape
Source: PLoS Comput Biol. 2025 Dec 3;21(12):e1013774. doi: 10.1371/journal.pcbi.1013774 (PMC12688157; doi:10.1371/journal.pcbi.1013774)
Supplement: Attachment — Submitted filename: BAGEL_Review_Response.pdf [file pcbi.1013774.s010.pdf]

Original comments by reviewers are in italics.
Our replies are in red.
Please note that references [] in this reply refer to the bibliography at the end of each question.

Reply to Reviewer #1

Both ESMFold and ESM-2 are well documented in terms of validation and accuracy, however, as a software article, results should show "Examples of biological problems solved using the software, including results obtained with the deposited test data and associated parameters." The four applications are rather demonstrative than solid ground applications. Results showed how the energetics of the models were improved, however, the authors do not provide sufficient biological insights to how these models could be validated or used. The work can benefit greatly by validation molecular dynamics simulations of the 10 main protein examples described. One of the applications targeting IDR is available as recent conference paper (reference 33), while another is in the form of preprint (reference 39). Another application citing Reference 38 is not relevant or not clear how it is connected to the text. In my opinion, one peer-reviewed published application would have been sufficient validation.

While we recognize the critique surrounding sufficient validation of the examples presented, what we wanted to show in this paper is examples of different design campaigns and how they can be seamlessly implemented using BAGEL's modular structure. Whether or not specific sequences will behave as designed strongly depends on the accuracy of the underlying predictors used, in this case, ESM-2 and ESMFold, not on the BAGEL package per se.

Both ESM2 and EMSFold, as the referee agrees, are well-validated against wet-lab experiments, and for the proof of principle provided here, we believe their high accuracy would translate, partially if not entirely, to our examples. Nevertheless, as the referee suggests, we have also already validated using molecular dynamics simulations most of the results shown here. Some specific examples are part of papers currently under review in two different articles already submitted for peer-review, and any reader can see and evaluate the *bona fide* and accuracy of our results on the arxiv versions, i.e., Ref. 33 and 39.

Having said that, to at least partially satisfy the reviewer's request, we now provide in the Supplementary Information the results of molecular dynamics (MD) runs not submitted elsewhere, showing the stability (at least on the timescale provided) of the designed systems. In [1,2], it was shown how even relatively short MD runs, much shorter than those presented here, are particularly useful to screen out proteins that correspond to unstable designs, and we expect a similar result to hold here.

We now make a reference to this validation in the main manuscript:

Nevertheless, to obtain an orthogonal validation of the stability of the designed sequences, as in [1, 2], we perform molecular dynamics trajectories, showing the structures are at least stable on the simulated timescales (details of all simulations are provided in the Supplementary Information III).

We omit any of this MD-based data from the main body of the paper in order to keep the narrative of the paper lean, and make the report concise for practical use (also considering we are above the maximum number of words for this type of manuscript).

[1] Merlicek, L.P., Neumann, J., Lear, A., Degiorgi, V., de Waal, M.M., Cotet, T.S., Mulholland, A.J. and Bunzel, H.A., 2025. Ai. zymes: A modular platform for evolutionary enzyme design. *Angewandte Chemie International Edition*, p.e202507031.
[2] Bunzel, H.A., Smith, J.A., Oliver, T.A., Jones, M.R., Mulholland, A.J. and Anderson, J.R., 2022. Photovoltaic enzymes by design and evolution. *bioRxiv*, pp.2022-12.

We have to keep in mind that deep learning prediction models are built around the notion of homology as guide for accuracy. In which case a model with long chain of bad pLDDTs will give bad results no matter how much it is mutated. The authors are already aware of this as described in their future outlook section. This work should be supported by sensitivity analysis that draws the reference line for overall accuracy (not only with reference to the starting model). For example, if a starting model is really bad, then the pipeline should be able to tell the user that quality is too low to proceed. To define the threshold for that, I propose a sensitivity analysis on a dataset of structures at various homology levels to the PDB.

While we agree with the reviewer that homology remains a strong guide for accuracy, and this surely remains the case for homology-based protein-folding algorithm, state-of-the-art deep-learning models such as ESMFold and AlphaFold2/3 still obtain relatively high TM-scores (average ~0.65) even when no MSA is present *at all* [3], showing that their accuracy is much less dependent on homology comparing to previous approaches to protein folding.
Possibly for this reason, when using these models, it is not true that “a long chain of bad pLDDTs will give bad results no matter how much it is mutated”. In fact (and possibly the reviewer misunderstood what we have written), we show that the opposite is true in some of the examples we discuss. For example, in the application where we show targeting of IDRs, which by their nature are chains of residues with low pLDDT, we see clearly that, through successive mutations, one *does* achieve a reduction in pLDDT, see also Figures 2a and 4c in the main body of the current manuscript.

In fact, it is a matter of current debate whether low pLDDT or PAE values really mean inaccurate predictions, or instead signal the presence of highly dynamical, disordered regions (in which case, the algorithm basically captures the fact that such sequences do not necessarily fold in any dominant structure, see [4]).

On a more general level, however, BAGEL can easily provide the user information about the expected accuracy of the designs. This is because some of the energy terms implemented in BAGEL, and typically used during optimization, e.g., the predicted Template Modelling score, the pLLDT and the PAE, *are* a measure of the confidence of the protein folding algorithm in its prediction. In protein design using deep-learning approaches, one often tries to generate structures that, regardless of other constraints, have low uncertainty, simply because these structures are better predicted and their experimental translation will be higher. In turn, this accuracy will be higher the closer the design sequence is to some homologous protein found in the protein folding model training database. By monitoring these metrics during optimization, the user can thus easily gauge how good their final design is. In all cases we studied and presented, either these metrics improve during optimization, or they are relatively higher compared to the beginning, and remain at values (>70) that have shown to be associated to good structural prediction [see for example Fig. 5 in Ref. 6], that is, predictions with low RMSD compared to experimentally determined structures.

To clarify the point above, we now make these points more explicitly in the text by adding the following sentences:

“Specifically, one can monitor the expected success of these designs by referring to the evolution of the confidence metrics, e.g., pLDDT, iPAE, or pTM, during optimization. As extensively shown (see [5,6]) by comparison between predictions and experiments, low values of the predicted confidence metrics strongly correlate to lower RMSD values between the predicted and experimentally determined structure, at least in the case of single-chain predictions for natural proteins. While a large-scale assessment of the translational accuracy of these metrics remains to be done for the case of designed proteins, the current available data show that high confidence metrics are indeed correlated to more successful designs, especially for the relevant case of protein binders.”

[3] Lin, Z., Akin, H., Rao, R., Hie, B., Zhu, Z., Lu, W., Smetanin, N., Verkuil, R., Kabeli, O., Shmueli, Y. and dos Santos Costa, A., 2023. Evolutionary-scale prediction of atomic-level protein structure with a language model. *Science*, *379*(6637), pp.1123-1130.

[4] Guo, H.B., Perminov, A., Bekele, S., Kedziora, G., Farajollahi, S., Varaljay, V., Hinkle, K., Molinero, V., Meister, K., Hung, C. and Dennis, P., 2022. AlphaFold2 models indicate that protein sequence determines both structure and dynamics. *Scientific reports*, *12*(1), p.10696.

[5] Akdel, M., Pires, D.E., Pardo, E.P., Jänes, J., Zalevsky, A.O., Mészáros, B., Bryant, P., Good, L.L., Laskowski, R.A., Pozzati, G. and Shenoy, A., 2022. A structural biology community assessment of AlphaFold2 applications. *Nature Structural & Molecular Biology*, *29*(11), pp.1056-1067.

[6] Hýskova, A., Maršálková, E. and Šimeček, P., 2025. Balancing Speed and Precision in Protein Folding: A Comparison of AlphaFold2, ESMFold, and OmegaFold. *bioRxiv*, pp.2025-06.

[7] Bennett, N.R., Coventry, B., Goreshnik, I., Huang, B., Allen, A., Vafeados, D., Peng, Y.P., Dauparas, J., Baek, M., Stewart, L. and DiMaio, F., 2023. Improving de novo protein binder design with deep learning. *Nature Communications*, *14*(1), p.2625.

I have reservations towards the use of the terms states and multi-states in the paper. These terms are commonly used in the protein 3D structure community to describe multi-state proteins. Multi-state proteins require very stringent and standardized definitions related often to activity or function. In the paper, multi-state was described in the narrow meaning of mutation-based states which were used for candidate generation via optimization, and that can be a source for confusion to the reader. This would sound as promising too much on the framework of general multi-state proteins. In my understanding, optimization is a heuristic approach that scans only a limited fraction of conformational space as opposed to other deterministic methods. While multiple optimizations can serve in sampling, they are not the ideal for multi-state predictions.

We believe we might not have been too clear in the text ourselves, as we are not using the term “multi-state” in any sense or form close to what the Reviewer is alluding to here.

Our definition of State here is purely a mathematical definition (and the name used to distinguish the associated Python class in the code): a State is simply a collection of interacting protein chains together with a set of energy terms applied to those chains. In the BAGEL code presented in the manuscript, a System is split into distinct (i.e., non-interacting) States, whose definition is required for calculating the loss function whose minimization gives our optimal protein sequence. For example, a 2-state system could be prepared by having one state (State 1) with chain A and B, and another state (State 2) that again contains chain B and a different chain C. Each of these two states is completely isolated from each other: in State 1, chain B only interacts with chain A, in State 2, B only interacts with C. However, the total energy of the

system is the sum of the energy of both States 1 and 2. Just to make an example of how to use States, if we want B to be a sequence that can bind to both protein A and protein C, we thus seek to optimize the total energy using energy terms whose reduction correlates to predicting binding to both sequences *in their separate states*.

To reiterate, a State in BAGEL simply means a collection of protein chains and associated energy terms, nothing more, it does not refer to a *conformational* state, the definition used by the Reviewer. In fact, although we do believe that BAGEL *could* be used to design such multi-conformational-state proteins with the correct choice of energy terms (and there are ongoing investigations in this regard in our group), we never make this claim here.

We now make this point more explicitly and clarify our definition by adding the following paragraph to the main text:

To avoid any confusion, we stress here that within this manuscript a State (with capital S) is always used only and exclusively with this specific definition: it is a set of single-chain proteins, together with its associated set of energy terms to be evaluated given predictions on these very same chains. It is not, therefore, a reference to a conformational state of such chains.

In this regard, we realise the misunderstanding might come from the fact that our optimization is not in conformational space, but in sequence space (we look for the protein sequence(s) that minimize the total energy). In this regard, as the referee correctly says, standard optimisation can sometimes get stuck in a local minimum, especially if one uses standard MCMC or steepest-descent methods. However, more advanced protocols, such as the simulated tempering procedure we use here (but there are also others, e.g, simulated annealing, already coded in BAGEL), help crossing energy barriers, strongly alleviating this issue. In fact, there are strong theoretical results *guaranteeing* convergence of these approaches to the global minimum.

The authors used several intuitive model quality and energetic evaluations (some of which are very recent, e.g. ones developed by Alphafold), however, why they have not included mainstream molecular mechanics computations of potential energy, which are widely used by researchers?

We appreciate the comment to include an additional energy term with a physics-based root, it is indeed something we have considered. In fact, we do mention this possibility in the Future Outlook section, highlighting that force-field based calculations would further expand the suite of Oracles, and will be prioritised in future releases. For the current, initial version of the BAGEL code, we have limited ourselves to terms that measure the quality of the design by the confidence metrics provided by folding oracles because, at least empirically, these terms already strongly correlate to experimental success, especially for the case of designed protein binders. It is also clear, however, that a combination of the confidence-based terms and physics-based terms will be the most useful, as recently show in Ref. [8]. For this reason, a Git-hub issue related to addition of physics-based energy terms has already been added on the BAGEL repository, and we will implement them in future releases. In this regard, having this manuscript published will also allow a larger community to converge around BAGEL, speeding up implementation of these additional features to our codebase.

[8] Nori, D., Parsan, A., Uhler, C. and Jin, W., 2025. BindEnergyCraft: Casting Protein Structure Predictors as Energy-Based Models for Binder Design. *arXiv preprint arXiv:2505.21241*.

How does this pipeline compare to FoldX? The FoldX program is known for its simple mutability engine to perform sequential point mutation screening (useful in protein-protein or protein-peptide interactions), followed by energetic scoring. It is known that beyond the single point mutants, the folding becomes less predictable with more mutations, especially for peptides.

A direct comparison between BAGEL and FoldX is not possible because they are very different tools, with different scopes.

FoldX is a well-established computational tool / force field for estimating protein energetics (in particular, changes in stability (ΔΔG) upon mutation), and for analyzing protein–protein or protein–DNA interactions. At its core, FoldX is thus an empirical energy function calibrated to experimental data; it's used for rapid evaluation of point mutations, scanning mutational effects. This is also why, as the reviewer correctly points out, accuracy in FoldX rapidly deteriorates when making multiple mutations.

However, FoldX is not a "design framework" supposed to find an optimal sequence for a specific task, it is an "energy model", that is, a tool to understand the effect of point mutation to physical (binding, stability) energies. In this regard, the energy reported by FoldX for different mutations is a physical energy, calculated by summing different contributions (electrostatics, van der Walls, hydrophobic,...).

Contrary to FoldX, BAGEL does not and cannot (at least not at present, see also previous question) determine the effect of point mutations on the thermodynamic stability of the folding of a sequence. BAGEL scope is to scan sequence space to find the protein sequence whose properties, as predicted by some "oracle" (e.g., a protein folding algorithm, a protein language model, or any function that maps a sequence to some other space) will minimize a loss function. In this regard, while one can scan sequence space by making point mutations, this is just one way to do it and any way of making mutations, e.g., combining different sequences, would also be possible and valid.

Unlike FoldX which has a single underlying energy model, BAGEL is model-agnostic, i.e., one can plug in different, multiple energy models that can be used at the same time, including the energy model of FoldX. In fact, crucially, the BAGEL's minimized loss is the sum of different terms that are not even necessarily energies in the physics/thermodynamic sense. They are instead energies in a broader, mathematical sense: scalar potentials bounded by below, whose value only depends on the current state of the system. Because of classical theorems related to Monte Carlo sampling, a total energy given by the sum of such terms means BAGEL's can converge to a well-defined solution.

To give a clear example, an energy term can measure how hydrophobic a sequence is, how close it is to a given template, or how well-predicted a protein folding algorithm says that sequence is. While in BAGEL we call these different terms "energy" terms, as common in the literature of energy-based optimization, we reiterate that they are not an energy at all in the stricter physics sense.

In summary, FoldX measures the effect of point mutations on physical energies related to binding and folding stability. BAGEL instead is a design algorithm that finds an optimal design by finding the minimum of a user-definable energy landscape made of heterogeneous terms.

We further clarify our definition of energy terms in the manuscript adding the following paragraph:

By construction, all BAGEL's energy terms are, in a broader mathematical sense, simply scalar potentials bounded by below. In other words, their value depends only on the current state of the system. However, these terms do not need to represent a physical energy in the thermodynamic sense - these physical energies are instead a more stringent subset of our energies. Importantly, as long as all terms are bounded scalar potentials, strong theoretical convergence results apply to Monte Carlo sampling.

Hydrophobic residues used in HydrophobicEnergy term do not seem to belong to a familiar hydrophobicity scale. Please justify why was Tyr or Cys or Ala or Gly not included?

We thank the Reviewer for making this comment, as it allows us to improve the clarity of our manuscript and highlight important features of BAGEL.

In defining a certain residue as Hydrophobic, we only included those that are generally considered as "strongly hydrophobic", therefore omitting Tyr, Cys, Ala and Gly which are all mildly hydrophobic according to various scales. While this choice is not uncommon (it was, for example, the same used by the team behind ESMFold), it can certainly influence the final design obtained.

Thanks to BAGEL's modularity and ease of implementation, a user can easily change this definition if necessary. In fact, one can also easily implement a HydrophobicEnergy term that differs from the current one, and be based on a continuous scale rather than the rough binary classification we use here. This is exactly one of the main advantages of BAGEL. In the revised text, we comment on this aspect adjusting the following sentences:

Hydrophobic residues are defined as valine, isoleucine, leucine, phenylalanine, methionine, and tryptophan, which are regarded as the most strongly hydrophobic. Users may, however, incorporate mildly hydrophobic residues such as tyrosine, cysteine, alanine, and glycine in their counting or, alternatively, implement custom terms that employ a more continuous hydrophobicity measure.

Minor:
Page 15: "a cheaper model" >>> "a computationally-cheaper model"
"an expensive model" >>> "a computationally-expensive model"

We have now also implemented these modifications in the revised manuscript.

Reply to Reviewer #2

1. Authors provide some beautiful examples, however, the efficiency was not mentioned. It would be better to show the system size, CPU/GPU counts and runtime for both per step and total. Furthermore, can BAGEL design large-size protein (e.g., 300 amino acids)? If so, how does runtime and resource usage (CPU/GPU scaling) compare for such cases?

We are thankful for this comment and the revised manuscript includes additional information on the computational costs. In general, the majority of the computational cost is paid at the

oracle-inference, therefore BAGEL is – within the context of the currently implemented Oracles – limited by GPU speed. While it is beyond the scope of the paper to extensively benchmark various protein oracles and their runtime with increasing system sizes, we instead refer the reader to previous work that has already done so [9], including the original ESMFold paper [10].

[9] Haley, O. C., Tibbs-Cortes, L., Hayford, R. K., Harding, S., Woodhouse, M., Cannon, E., … Andorf, C. (2025). Why do some predicted protein structures fold poorly? Benchmarking AlphaFold, ESMFold, and Boltz in maize. doi:10.1101/2025.07.05.663230
[10] Lin, Z., Akin, H., Rao, R., Hie, B., Zhu, Z., Lu, W., Smetanin, N., Verkuil, R., Kabeli, O., Shmueli, Y. and dos Santos Costa, A., 2023. Evolutionary-scale prediction of atomic-level protein structure with a language model. Science, 379(6637), pp.1123-1130.

2. For peptide or epitope binder design, it would be helpful to see a comparison against other AI-based models (e.g., RFdiffusion) to test BAGEL's accuracy and performance within the current landscape of state-of-the-art methods.

We appreciate the Reviewer's comment. We agree that testing BAGEL's accuracy against other methods, such as the RF-Diffusion-based one, or BindCraft, would be beneficial. Nevertheless, BAGEL is a model-agnostic framework that can be applied to a much wider set of problems than any of the aforementioned pipelines. We thus find it difficult to specify a protein design problem, where comparing such methods would be fair. Lastly, it would require extensive experimental validation, which we considered outside of the scope of this article describing a software package. Otherwise, we would be limited to comparing based on *in silico* metrics, where BAGEL would perform well by design, as we show that the simulated tempering minimization is able to find minima with values of these confidence metrics below thresholds usually considered sufficient for subsequent wet-lab testing.

Reply to Reviewer #3

We thank the Reviewer for an overall positive response.

While we make an effort to reply to the suggested improvements, we notice that most if not all have been likely generated by a large language model (see the attached analysis by GPTZero, which claims that 99% of the text is AI-generated).

In fact, many comments are irrelevant, with a clear misunderstanding of the paper content -or showing the paper has not been read in full. Replying to ChatGPT-generated questions is not particularly productive to the academic discourse or the peer-review process, but we still take the chance, at least for some that are pertinent, to improve our manuscript.

1. The authors must include brief benchmarking results or discussion on runtime scaling, particularly when using large oracles such as AlphaFold or protein language models.

Inference time benchmarking has been previously done in the original ESMFold manuscript [DOI: 10.1126/science.ade2574].

2. The authors should present or reference evaluation metrics (e.g., stability scores, binding energy estimates, or structural RMSDs) in order to better contextualize BAGEL's performance relative to established baselines.

This comment completely misunderstands the nature of our paper. Experimental validation, such as comparing RMSDs to experimentally-derived structures, is entirely beyond the scope of a publication focused on describing a software package. As an orthogonal validation, we show binding free energy estimates in the cited manuscript on targeting of IDRs. Moreover, BAGEL by design will succeed in finding sequences with good *in silico* metrics such as stability scores, binding energy estimates or structural RMSDs (unclear to what we would compare to, or whether the Reviewer asks for crystallizing the designs experimentally), as those can be integrated by the user as a specific energy term.

3. Discuss integration and interoperability. basically, explain how BAGEL can complement or interface with other design ecosystems (e.g., Rosetta, PyRosetta, or AlphaFold-Multimer) for broader adoption.

We better describe the role of BAGEL in the broader design ecosystem in the revised manuscript by adding the following sentence:

We note, however, that even with continued development, BAGEL does not constitute a plug-and-play package to any protein design problem, and should be used with a wider set of computationally available tools, such as PESTO~\cite{Krapp_2023} to find binding sites, or sequence inpainting models \cite{Dauparas2022, Goverde2024, Dauparas2025} to increase protein candidate diversity. In the long-term, on the other hand, all these could in principle be employed within the BAGEL framework, as a part of a broader protein design optimization campaign.

4. Expand briefly on planned or ongoing experimental validations to strengthen confidence in the practical impact of BAGEL-generated designs.

We are currently validating three applications experimentally: a) binders to CD22 (not a part of the manuscript), b) binders to IDRs (alpha-synuclein), and c) enzyme variants. We do not discuss this further in the manuscript to keep the paper focused. As stated in the paper and the replies above, our paper focuses only on the software package, and the designs are inherently limited by the accuracy of the Oracles. Improving these oracles is clearly also outside of the scope of the manuscript, but we mention in passing that improved oracles will make all BAGEL's design also better from a translational point of view.

5. Condense some descriptive passages and emphasize unique algorithmic contributions, ensuring that conceptual novelty remains at the forefront.

There are no “unique algorithmic contributions” in BAGEL: it is a modular framework whose main goal is to allow the user to easily combine many different, heterogeneous (in output) algorithms together, and use them to build arbitrary complex loss functions, further automatising their minimisation (or their sampling).
In practice, it is a software that allows to restate any protein design problem into building and optimising a loss function, and quickly check the result of different choices, allowing quick iteration over multiple designs.

6. Given that AlphaFold2/3 produce high-accuracy static structures, how does the lack of conformational ensemble modeling affect the reliability of designs that depend on flexibility or induced fit?

BAGEL is limited by the conformational ensemble accuracy of whatever folding oracles is used. We do not discuss the problem of conformational ensemble sampling, as that is not the focus of the package, nor of the paper. If AlphaFold2 or AlphaFold3 only provide static structures, it simply means they should not be used as oracles for designing protein whose function strongly depends on sampling conformations - but this is a user choice. While we notice in passing that we never use AlphaFold2/3 in any of our examples, so it is not even clear why this question would be relevant (as we suspect, a GPT and not a human), we still take this chance to make these points about oracles as clear as possible in the paper, by adding the following sentence:

Eventually, whether or not a design will work in practice depends on two important aspects: the quality and accuracy of the Oracles used, and whether the design problem is well-described by the loss function provided by the user. Just to make an example, if a functional property of a protein is strongly dependent on its structural fluctuations, an Oracle able to evaluate these effects should be used. Failure to use the right Oracle, or failure to account for these effects in the loss function, will necessarily result in an experimentally unsuccessful design. In this regard, the continuous improvements of the community around different models, i.e., Oracles, as well as better understanding of their use and limitations, will allow users to use BAGEL better. In other words, while BAGEL allows to easily translate any design problem into a loss function, and quickly test the results of its minimization, it does not replace expert knowledge, which should be used as much as possible to pre-condition the design problem to help finding the correct solution.

7. Could integrating molecular dynamics or normal mode analyses into the energy evaluation improve BAGEL's ability to capture dynamic effects?

Yes. We already exactly mention this point in the Future Outlook section on using MD as a potential oracle.

8. How sensitive is BAGEL's optimization to cases where small conformational shifts dramatically alter binding energetics?

The current implementation of BAGEL does not include any physics-based binding energetics as part of its energy terms suite. BAGEL's modular framework allows the user to both a) add any optimization algorithm of their liking, and b) implement any algorithm that computes such binding energetics. Therefore, BAGEL itself is not inherently limited, or poorly sensitive to conformational shifts. This will be only a function of the exact minimization and energetics algorithm employed.

9. To what extent do pLDDT, pTM, or iPAE-based loss terms bias optimization toward regions of the predictor's uncertainty rather than true physical minima

In our optimization, the exact opposite happens here. In the majority of our applications (apart from selective design where we discourage off-target binding), the goal is to minimize uncertainty, which (under the assumptions of perfect Oracles) yields successful sequence candidates. Nevertheless, it is true that regions of low uncertainty (i.e. high predictor's confidence) might not necessarily lead to true physical minima in the free-energy landscape of folding, it is the underlying assumption of all current protein folding algorithms employed for computational protein design.

To which extent this occurs depends on the influence of those terms in the loss function, *if* they are used at all in its definition. Thus, it depends on a user-defined choice. It is widely accepted

as good practice to introduce such terms as energies to penalise results that are not predicted to be accurate when using protein-folding oracles, but, again, it's the user that specifies this depending on how the loss function is built.

10. Are there strategies in BAGEL to decouple model confidence from energy evaluation for e.g., through ensemble averaging, reweighting, or uncertainty calibration?

The energy evaluation in BAGEL is purely a mathematical construct. It can be derived from any Oracle's output a user can think of. Thus, by definition, if one uses a confidence metric as an energy term in BAGEL, the model confidence cannot be decoupled from computing the System or State energies. Only when not using a model confidence as an energy term is the System energy independent of the Oracle's confidence.

11. How might BAGEL handle inconsistent confidence across multiple states or chain interfaces?

Again, it seems as though the question does not understand the idea of a model's 'confidence'. In terms of confidence across multiple States, this would be taken care of during the energy minimization itself, and would roll out according to the energy landscape and traversing in the energy landscape, as is defined by the user in the System energy function. It is unclear what is meant by inconsistent confidence. The confidence can differ across different States, which is (again) a feature, and not a bug. For instance, in the selective binding application example, a lower confidence in the structure for the off-target is utilized to optimize binding for the target protein, while reducing interactions with the off-target protein. In terms of chain interfaces, it is unclear what the question refers to here. Confidence across a chain interface is usually correlated with interface Predicted Aligned Error — where its 'inconsistency' across two States is again employed in the off-target example to achieve selective binding.

12. How does BAGEL handle regions where the oracle's confidence is low? Are there fallback strategies or uncertainty-aware loss terms?

The confidence of a folding oracle is directly used as an energy term. The goal of BAGEL, and many deep learning-based design pipelines, is to find a sequence that, upon folding, has a high confidence in the structure. For that matter, in the context of optimization in the search of a candidate sequence with some desired properties, the confidence metric is a useful feature, and not a bug or a problem. This aspect is clearly misunderstood in the question, and there are no fallback strategies because it is not a problem.

13. Can the framework incorporate experimentally measured kinetic or thermodynamic data to guide multi-state optimization?

If such data is incorporated into a predictive Oracle, then one can use it to drive the optimization process, as is suggested in the Future Outlook. For example, one could use FoldX (see also previous response to Ref 1) to provide a penalty to mutations that decrease stability.

14. What is the computational cost of large-scale multi-state runs compared with single-state Monte-Carlo pipelines?

As previously discussed (Ref 2, question 1), this depends on the computational cost of individual Oracles (already discussed elsewhere), and then the respective sequence lengths (i.e., system sizes). We do not further comment, as the question is very vague and does not refer to any specific part of BAGEL. For instance, it is not even clear what the Reviewer means by ‘single-state Monte-Carlo pipelines’ (more precisely, what is a “single-state pipeline”?)

15. How will future diffusion-based or language-model predictors (e.g., AlphaFold-3) be integrated without redesigning the oracle interface?

This is a great question, but it is already tackled in the Future Outlook, where we very clearly mention that the Oracle interface, and the boileroom backend package, have been written in such a way as to be model-agnostic, treating Oracles as effective black boxes. Including other Oracles is in ongoing development as we speak. Moreover, we note that this question is inaccurate, as AlphaFold3 is not based on a language model (further suggesting it is AI-generated..).

16. How does BAGEL’s multi-state formalism handle coupling between shared chains across different states without double-counting energies?

The question does not seem to understand the definition of an energy, which is fundamentally only a terminology employed to define a design constraint, i.e., a loss that needs to be minimized.
If the same Chain object belongs to different States, it only means that each State has a protein with a specific sequence, and this sequence will remain the same for different States objects every time a mutation is made - something handled easily by copying Chain objects into different States by reference.
Whether or not a specific energy term should be counted in different states is something the user must define (by defining the State itself). In some cases, this will be necessary but it would be a feature, not a bug or a problem. For example, the user might want to have an energy term that is associated with how well a protein structure is defined. This term can be present in different states, and does not even have to evaluate to the same number for different States, considering that the predicted accuracy for a prediction depends on all chain presents, so it can vary for the same sequence if associated with other, differing chains in the same State.

17. The additive N-body energy formulation mirrors molecular mechanics approaches. How are weights w !"calibrated to avoid dominance by any single energy term?

The user chooses the weights based on their protein design task. For our applications shown, these are chosen by trial and error. These weights effectively determine the features of the energy landscape, and the user should use that information accordingly. Moreover, each energy term is normalized accordingly to ensure different energy terms span similar magnitudes, between 0 to 1 (for those that are not, users can compensate with an appropriate weight). For instance, the Predicted Aligned Error when directly retrieved from the Oracle takes up values from 0 to 30, while, in BAGEL, we normalize to 0 to 1. We will include better guidance surrounding the practical use in the online documentation of the package.

18. Can BAGEL serve as a benchmarking framework to evaluate the robustness of new oracles or differentiable predictors?

BAGEL does not support differentiable predictors. Rather than BAGEL, the backend package boileroom could be used to unify access to various protein structure Oracles, and thus provide a

benchmark suite for their evaluation. Although a valuable point, we do not consider it relevant to the topic of the work presented here.

19. The notation is occasionally overloaded or ambiguous. For instance, the use of {C}S and {C} could be more consistently distinguished ; sometimes {C}S refers to chains in a particular state, and sometimes to their sequences.
a. Equation (1) defines Ω = ({C}, {S}), which conceptually conflates the system and its states. A more precise statement might specify Ω as a set of states, each defined by its constituent chains and energy functions: Ω = {S# = ({C}$! , E$!)}
b. Summation indices (e.g., over S, j, or s) should be clearly delimited; currently, they shift between symbolic and index notation.

A system is a <u>collection</u> of states, so the notation is not overloaded, nor it conflates system and states as they are different objects (both mathematically and from a programming perspective). The reviewer did not likely understand the definition of a State and a set of Chains (sequences). A state *is* a set of chains together with an associated energy function specifically evaluated over that state (it is not, instead, a conformational state). A system can be built by joining different states together (each with its own, unique energy function). Finally, summation indices are always explicitly provided, and we never shift between symbolic and index (Einstein) notation to specify sums.

20. While defining three types of oracles (folding, embedding, and property) is comprehensive, the notation for outputs (e.g., y, z, r) could be standardized. It would help to specify how these oracle outputs are integrated into ε; are ε functions differentiable ? Or do they operate as heuristic scoring functions? This distinction affects both theoretical guarantees and computational tractability.

The notation is not unified exactly to distinguish between different types of Oracles that could be theoretically employed. The oracle outputs are integrated into energy terms through their respective implementations in Section 3.2 on Energy Terms. That is, the energy terms are functions of the oracle outputs. Neither the oracles, nor the energy terms have to be differentiable, as specifically mentioned in the Abstract. Energy terms are mathematical abstractions to define a minimization over an energy landscape, thus – in practice – they could be viewed as heuristic scoring functions, rather than a physical energy, which the Reviewer might be alluding to here.

*21.The MCMC section could more explicitly describe the mutation operator M**,** e.g., whether it performs single-residue substitutions, insertions/deletions, or compositional sampling across multiple chains.c. d. The temperature schedule T % is mentioned but not defined; providing a concrete annealing scheme (e.g., exponential or logarithmic) would make the procedure more actionable. The convergence or stopping criteria ("system's energy reaches a plateau") could be better formalized, perhaps via a threshold on ΔE variance or effective sample size.*

The mutation operator, specifically then the implementation of the MutationProtocol, is clearly described in Section 3.3. The temperature schedule is clearly described in Section 3.4 on Minimizers. There is no stopping criteria, i.e., the optimization runs for a user-defined number of steps, but a user can simply define their own, related to any specific metric (and/or an energy term), such as iPAE, pLDDT or pTM, whose evolution BAGEL can easily monitor. Same thing for the temperature schedule. Pre-coded in BAGEL we have simulated annealing and simulated tempering because they are often used as minimizers, but the possibility to use a completely arbitrary temperature schedule is actually the default, and the user can choose any schedule

they want, and easily implement it using simple Python instructions.

In other words, we let the user define how to do the minimisation, and make it possible to implement almost any choice in a very easy way thanks to the modularity of the code. We leave this freedom because such choices are invariably problem-specific, and is determined by the user's design goals.

22. Some equations (especially Eq. 2–4) could be formatted more clearly with alignment and consistent subscripts.

We are unclear what is meant by Equations 2-4, as there is no Equation 4. In the revised manuscript, we added numbering to all Equations as we were missing that in the original draft.

23. What mechanisms are in place (documentation, API standardization, model registry) to facilitate external contributions and ensure reproducibility?

We include a Zenodo repository with a time-capsuled version of the codebase (https://zenodo.org/records/15808839), including the scripts to qualitatively reproduce the results of the exemplary designs. It is beyond the scope of the paper to go into the details of the backend package boileroom (https://github.com/softnanolab/boileroom), which unifies the API of different Oracles. Users are welcome to explore boileroom's open-source GitHub repository themselves. Otherwise, BAGEL is actively being developed in the open on GitHub (https://github.com/softnanolab/bagel). We specifically address contributions in the text already:

We aim to cultivate a vibrant community around BAGEL by encouraging contributions via GitHub pull requests.

FAQs

What is GPTZero?

GPTZero is the leading AI detector for checking whether a document was written by a large language model such as ChatGPT. GPTZero detects AI on sentence, paragraph, and document level. Our model was trained on a large, diverse corpus of human-written and AI-generated text with support for English, Spanish, French, German, and other languages. To date, GPTZero has served over 10 million users around the world, and works with over 100 organizations in education, hiring, publishing, legal, and more.

When should I use GPTZero?

Our users have seen the use of AI-generated text proliferate into education, certification, hiring and recruitment, social writing platforms, disinformation, and beyond. We've created GPTZero as a tool to highlight the possible use of AI in writing text. In particular, we focus on classifying AI use in prose. Overall, our classifier is intended to be used to flag situations in which a conversation can be started (for example, between educators and students) to drive further inquiry and spread awareness of the risks of using AI in written work.

Does GPTZero only detect ChatGPT outputs?

No, GPTZero works robustly across a range of AI language models, including but not limited to ChatGPT, GPT-5, GPT-4, GPT-3, Gemini, Claude, and AI services based on those models.

What are the limitations of the classifier?

The nature of AI-generated content is changing constantly. As such, these results should not be used to punish students. We recommend educators to use our behind-the-scene Writing Reports as part of a holistic assessment of student work. There always exist edge cases with both instances where AI is classified as human, and human is classified as AI. Instead, we recommend educators take approaches that give students the opportunity to demonstrate their understanding in a controlled environment and craft assignments that cannot be solved with AI. Our classifier is not trained to identify AI-generated text after it has been heavily modified after generation (although we estimate this is a minority of the uses for AI-generation at the moment). Currently, our classifier can sometimes flag other machine-generated or highly procedural text as AI-generated, and as such, should be used on more descriptive portions of text.

I'm an educator who has found AI-generated text by my students. What do I do?

Firstly, at GPTZero, we don't believe that any AI detector is perfect. There always exist edge cases with both instances where AI is classified as human, and human is classified as AI. Nonetheless, we recommend that educators can do the following when they get a positive detection: Ask students to demonstrate their understanding in a controlled environment, whether that is through an in-person assessment, or through an editor that can track their edit history (for instance, using our Writing Reports through Google Docs). Check out our list of several recommendations on types of assignments that are difficult to solve with AI.

Ask the student if they can produce artifacts of their writing process, whether it is drafts, revision histories, or brainstorming notes. For example, if the editor they used to write the text has an edit history (such as Google Docs), and it was typed out with several edits over a reasonable period of time, it is likely the student work is authentic. You can use GPTZero's Writing Reports to replay the student's writing process, and view signals that indicate the authenticity of the work.
See if there is a history of AI-generated text in the student's work. We recommend looking for a long-term pattern of AI use, as opposed to a single instance, in order to determine whether the student is using AI.

AI Report

We are highly confident this text is

AI Generated

AI Probability

99%

This number is the probability that the document is AI generated, not a percentage of AI text in the document.

Plagiarism

?

The plagiarism scan was not run for this document. Go to gptzero.me to check for plagiarism.

Review-SA-BAGEL.pdf - 10/11/2025

Jakub Lala

BAGEL: Protein Engineering via Exploration of an Energy Landscape

This manuscript presents BAGEL, a thoughtfully designed, modular framework that redefines computational protein design as a problem of energy landscape exploration rather than purely gradient-based optimization. The work is timely and relevant, addressing long-standing limitations in differentiable, model-specific design workflows. By integrating multi-state optimization, nondifferentiable objective handling, and flexible oracle integration, BAGEL represents a substantial conceptual and practical advancement toward a more general, programmable paradigm for protein engineering.

The manuscript succeeds in clearly articulating the motivation and theoretical foundations of BAGEL, as well as demonstrating its versatility across diverse applications from peptide binder design to enzyme variant generation. The model-agnostic philosophy and open-source accessibility are notable strengths, positioning BAGEL as a forward-compatible platform that can evolve alongside emerging deep learning models and molecular predictors.

To further strengthen the work, the following improvements are suggested:

The authors must include brief benchmarking results or discussion on runtime scaling, particularly when using large oracles such as AlphaFold or protein language models.

The authors should present or reference evaluation metrics (e.g., stability scores, binding energy estimates, or structural RMSDs) in order to better contextualize BAGEL's performance relative to established baselines.

Discuss integration and interoperability. basically, explain how BAGEL can complement or interface with other design ecosystems (e.g., Rosetta, PyRosetta, or AlphaFoldMultimer) for broader adoption.

Expand briefly on planned or ongoing experimental validations to strengthen confidence in the practical impact of BAGEL-generated designs.

Condense some descriptive passages and emphasize unique algorithmic contributions, ensuring that conceptual novelty remains at the forefront.

Given that AlphaFold2/3 produce high-accuracy static structures, how does the lack of conformational ensemble modeling affect the reliability of designs that depend on flexibility or induced fit?

Could integrating molecular dynamics or normal mode analyses into the energy evaluation improve BAGEL's ability to capture dynamic effects?

How sensitive is BAGEL's optimization to cases where small conformational shifts dramatically alter binding energetics?

To what extent do pLDDT, pTM, or iPAE-based loss terms bias optimization toward regions of the predictor's uncertainty rather than true physical minima

Are there strategies in BAGEL to decouple model confidence from energy evaluation for e.g., through ensemble averaging, reweighting, or uncertainty calibration?

How might BAGEL handle inconsistent confidence across multiple states or chain interfaces?

How does BAGEL handle regions where the oracle's confidence is low? Are there fallback strategies or uncertainty-aware loss terms?

Can the framework incorporate experimentally measured kinetic or thermodynamic data to guide multi-state optimization?

What is the computational cost of large-scale multi-state runs compared with single-state Monte-Carlo pipelines?

How will future diffusion-based or language-model predictors (e.g., AlphaFold-3) be integrated without redesigning the oracle interface?

How does BAGEL's multi-state formalism handle coupling between shared chains across different states without double-counting energies?

The additive N-body energy formulation mirrors molecular mechanics approaches. How are weights w_{j s} calibrated to avoid dominance by any single energy term?

Can BAGEL serve as a benchmarking framework to evaluate the robustness of new oracles or differentiable predictors?

The notation is occasionally overloaded or ambiguous. For instance, the use of \{\mathrm{C}\} \mathrm{S} and \{\mathrm{C}\} could be more consistently distinguished; sometimes \{\mathrm{C}\} \mathrm{S} refers to chains in a particular state, and sometimes to their sequences.

a. Equation (1) defines \Omega=(\{\mathrm{C}\},\{\mathrm{S}\}), which conceptually conflates the system and its states. A more precise statement might specify \Omega as a set of states, each defined by its constituent chains and energy functions:

\Omega=\left\{S{i}=\left(\{C\}{S{i}}, E{S_{i}}\right)\right\}

b. Summation indices (e.g., over \mathrm{S}, \mathrm{j}, or s) should be clearly delimited; currently, they shift between symbolic and index notation.

While defining three types of oracles (folding, embedding, and property) is comprehensive, the notation for outputs (e.g., y_{s}, \vec{z}, r) could be standardized. It would help to specify how these oracle outputs are integrated into \varepsilon; are \varepsilon functions differentiable? Or do they operate as heuristic scoring functions? This distinction affects both theoretical guarantees and computational tractability.

21.The MCMC section could more explicitly describe the mutation operator M, e.g., whether it performs single-residue substitutions, insertions/deletions, or compositional sampling across multiple chains.

c. The temperature schedule T_{n} is mentioned but not defined; providing a concrete annealing scheme (e.g., exponential or logarithmic) would make the procedure more actionable.

d. The convergence or stopping criteria ("system's energy reaches a plateau") could be better formalized, perhaps via a threshold on \Delta \mathrm{E} variance or effective sample size.

Some equations (especially Eq. 2-4) could be formatted more clearly with alignment and consistent subscripts.

What mechanisms are in place (documentation, API standardization, model registry) to facilitate external contributions and ensure reproducibility?

Sentences that are likely AI-generated.
